# Physiological Effects of 2-Bromoethanesulfonate on Hydrogenotrophic Pure and Mixed Cultures

**DOI:** 10.3390/microorganisms10020355

**Published:** 2022-02-03

**Authors:** Washington Logroño, Marcell Nikolausz, Hauke Harms, Sabine Kleinsteuber

**Affiliations:** Department of Environmental Microbiology, Helmholtz Centre for Environmental Research–UFZ, 04318 Leipzig, Germany; washington.logrono@ufz.de (W.L.); marcell.nikolausz@ufz.de (M.N.); hauke.harms@ufz.de (H.H.)

**Keywords:** formic acid, methanogenesis, homoacetogenesis, acetic acid, formate dehydrogenase, formate synthase, power to gas, biological biogas upgrading, biomethane

## Abstract

Mixed or pure cultures can be used for biomethanation of hydrogen. Sodium 2-bromoethanesulfonate (BES) is an inhibitor of methanogenesis used to investigate competing reactions like homoacetogenesis in mixed cultures. To understand the effect of BES on the hydrogenotrophic metabolism in a biomethanation process, anaerobic granules from a wastewater treatment plant, a hydrogenotrophic enrichment culture, and pure cultures of *Methanococcus maripaludis* and *Methanobacterium formicicum* were incubated under H_2_/CO_2_ headspace in the presence or absence of BES, and the turnover of H_2_, CO_2_, CH_4_, formate and acetate was analyzed. Anaerobic granules produced the highest amount of formate after 24 h of incubation in the presence of BES. Treating the enrichment culture with BES led to the accumulation of formate. *M. maripaludis* produced more formate than *M. formicicum* when treated with BES. The non-inhibited methanogenic communities produced small amounts of formate whereas the pure cultures did not. The highest amount of acetate was produced by the anaerobic granules concomitantly with formate consumption. These results indicate that formate is an important intermediate of hydrogenotrophic metabolism accumulating upon methanogenesis inhibition.

## 1. Introduction

Power to Gas (P2G) refers to the storage of surplus electricity from renewable energies as a combustible gas [1,2]. Hydrogen is produced through water electrolysis and can be used directly or fed to a methanation process [2]. Methanation of hydrogen can be a thermochemical or biological process (biomethanation of hydrogen) [1]. Biomethanation of hydrogen as a biological biogas upgrading technology integrates well with the existing anaerobic digestion (AD) technology or is used as a standalone carbon capture and utilization technology [2]. Biogas upgrading methods [3,4,5] as well as the hydrogen-assisted pathways [6] have been reviewed. Biological biogas upgrading based on hydrogen biomethanation makes use of in situ (biocatalysis in the main anaerobic digester), ex situ (biocatalysis in a reactor other than the main anaerobic digester) or hybrid (combining in situ and ex situ) concepts [4,7,8]. Biomethanation of hydrogen converts the CO_2_ content of biogas into additional methane via the CO_2_-reductive pathway of hydrogenotrophic methanogens. The conversion of hydrogen and CO_2_ to methane is carried out by pure cultures of hydrogenotrophic methanogens [9,10] or mixed cultures [4]. Mixed cultures have certain economic and process advantages over pure cultures [4,11]. However, competing reactions, such as homoacetogenesis, are difficult to control. When performing biomethanation of hydrogen with mixed cultures, hydrogenotrophic methanogenesis (Equation (1)), homoacetogenesis (Equation (2)), syntrophic acetate oxidation (SAO) (Equation (3)), and acetoclastic methanogenesis (Equation (4)) can be expected to take place. The formate-hydrogen interconversion (Equation (5)) is also possible. Equations showing these reactions are given below with Gibbs free energy (ΔG°′) values according to [12]

(1) $4H_{2}+{\mathrm{HCO}}_{3}^{-}+{\;H}^{+}\;\to {\mathrm{CH}}_{4}+3H_{2}O$        ΔG°′ = −135.6 kJ/reaction
(2) $4H_{2}+2HCO_{3}^{-}+H^{+}\;\to CH_{3}COO^{-}+4H_{2}O$  ΔG°′ = −104.6 kJ/reaction
(3) $CH_{3}COO^{-}+4H_{2}O\;\to 2HCO_{3}^{-}+H^{+}+4H_{2}$  ΔG°′ = +104.6 kJ/reaction
(4) $CH_{3}COO^{-}+H_{2}O\;\to HCO_{3}^{-}+CH_{4}$       ΔG°′ = −31.0 kJ/reaction
(5) $H_{2}+HCO_{3}^{-}\;\to HCO_{2}^{-}+H_{2}O$          ΔG°′ = −1.3 kJ/reaction

Previous biomethanation studies found acetate formation after hydrogen injection [13,14,15]. Stable isotope experiments during biomethanation of hydrogen revealed that 61 ±3% of the injected hydrogen was consumed via homoacetogenesis, and the highest acetate concentration was observed at CO_2_ concentrations lower than 7%, especially during the initial hydrogen injections [13]. However, repeated hydrogen injections fueled hydrogenotrophic methanogenesis rather than homoacetogenesis [13]. Recently, we demonstrated that selective methane production with a hydrogenotrophic enrichment culture can be achieved by an adequate media composition, especially using sodium sulfide instead of cysteine as reducing agent. Thereby, methane contents that comply with grid standards (>97%) could be achieved [16]. However, hydrogen is also used as an electron donor to build up microbial biomass. A previous study showed that 8.6–14.7% of the hydrogen fueled biomass formation [17].

It is known that formate is an alternative electron donor for hydrogenotrophic methanogens in natural as well as in engineered systems [18]. It could thus be speculated that formate plays a role during H_2_/CO_2_ biomethanation for biogas upgrading. A previous study showed that pure cultures of methanogens or acetogens were capable of transiently producing formate when they were fed with H_2_/CO_2_ as long as they were able to use both hydrogen and formate for methanogenesis or homoacetogenesis [19]. It is worth noticing that *Methanosarcina barkeri,* which is often found in AD systems, possesses a gene cluster encoding formate dehydrogenase, which suggests its ability to use formate [20]. Recently, in co-culture experiments with *Clostridium cellulovorans* 743B where formate was also an intermediate, *M. barkeri* was reported to perform methanogenesis from all intermediates, i.e., H_2_, formate, and acetate [21]. Moreover, it is known that the acetogenic bacterium *Acetobacterium woodii* produces formate from H_2_/CO_2_ [22,23]. In the AD reaction cascade, bacteria produce formate and H_2_ and rely on methanogenic partners to keep these intermediates at sufficiently low levels to make the reaction thermodynamically feasible [12,24,25,26].

The use of specific methanogenesis inhibitors allows investigating other metabolic functions than methanogenesis (e.g., homoacetogenesis or sulfate reduction) in mixed anaerobic cultures [27]. Most frequently used inhibitors are 2-bromoethanesulfonate (BES), 2-chloroethanesulfonate (CES), 2-mercaptoethanesulfonate (MES), and lumazine. The chemical structures of these compounds are analogous to coenzyme M and inhibit the methyl transfer reaction in methanogens to produce methane [28]. In addition, ethylene, acetylene, and other unsaturated hydrocarbons showed potential for application as methanogenesis inhibitors [29,30,31,32].

Although formate synthesis during methanogenesis inhibition with BES was demonstrated for pure culture methanogens and sewage sludge in the early 1990s [33], this process was not discussed in later literature. In anaerobic bioreactor sludge, acetate synthesis from H_2_/CO_2_ in the presence of BES was demonstrated [34], but the authors did not discuss the possible involvement of formate. Another study reported high concentration of acetate during fed-batch H_2_ supply operation in the absence of BES, however, formate was neither reported nor discussed [35]. A recent study investigating the competition between homoacetogenesis and hydrogenotrophic methanogenesis in sludge samples showed that in the presence of BES, formate was transiently produced and consumed [36], but clear trends were not observed, likely due to the sampling time interval. Another study showed simultaneous biogas upgrading and acetate production when using BES as a methanogenesis inhibitor; however, the authors did not observe the involvement of formate in the process likely because it was consumed at a fast rate [17]. The two aforementioned studies thus did not report formate in their experiments.

A coupled biosynthesis of value-added chemicals and biogas upgrading in one bioprocess was recently demonstrated by using BES [17]. Other studies have used BES in bioelectrochemical systems to inhibit methanogenesis and promote bioelectrosynthesis of value-added chemicals [31,37,38,39]. However, formate production has not been reported so far and deserves further investigation. The use of methanogenesis inhibitors in such electrochemical systems might facilitate electron allocation to target molecules such as hydrogen, acetate, or higher carbon molecules.

In biomethanation of hydrogen with mixed cultures (either ex situ or in situ), homoacetogenic bacteria are also enriched besides hydrogenotrophic methanogens, and acetate appears as the main side-product [4,35,40,41]. Mohd Yasin et al., measured low concentrations of formate at the start of hydrogen biomethanation, which could be derived from the inoculum since it was not further detected during the incubation period with H_2_/CO_2_ [42]. However, we recently observed that formate was produced and consumed during non-inhibited hydrogen biomethanation, indicating that formate is a common intermediate in methanogenic communities [16]. However, it is not clear whether this intermediate can be attributed to the methanogenic or to the homoacetogenic metabolism. Here, we investigated the effect of BES on methanogenic pure cultures, a hydrogenotrophic enrichment culture, and crushed anaerobic granules from a paper wastewater treatment plant to determine the formate production and consumption dynamics during hydrogenotrophic growth under methanogenesis inhibition and non-inhibition conditions.

## 2. Materials and Methods

### 2.1. Chemicals, Media, and Cultivation Conditions

2-Bromoethanesulfonic acid sodium salt (BES) with a purity of 98% was purchased from Sigma Aldrich. All chemicals used in this study were of the highest purity available. Strict anaerobic techniques were used throughout the study. The headspace/liquid volume ratio was set to three for all cultivation experiments independently of the bottle volume. The headspace of all culture bottles was flushed with H_2_/CO_2_ (80/20%) and pressurized to 2.2 bar absolute after inoculation as previously described [16]. The culture bottles were incubated at 37.3 °C with orbital shaking (200 rpm). All experiments were conducted in 3–5 biological replicates based on previously observed variation among replicates. Inhibition experiments were done at a final BES concentration of 50 mM, a concentration that has been used in other studies [17,38].

The major features of each cultivation medium are shown in Table 1. The media composition is detailed in the Supplementary Materials (Tables S1–S7). Media A, A1, and A2 are modified versions of the mineral medium DSMZ1036 (https://bacmedia.dsmz.de/medium/1036 (accessed on 16 December 2021)); detailed composition and modifications were described elsewhere [16]. Medium B is a mineral medium, which was described in a previous study [43]. Medium C is a mineral medium as reported earlier [44].

### 2.2. Inhibition Experiments with Anaerobic Granules

Fresh anaerobic granules were collected from a paper industry wastewater treatment plant, transported to the laboratory under a nitrogen atmosphere and incubated overnight under mesophilic conditions (38 °C). The inoculum preparation followed the procedures described in our previous study [45], with minor modifications. First, a composite sample (granules of all sizes and wastewater) was taken and pestled under a nitrogen stream (the process lasted less than 30 min). Secondly, 100 g of the crushed granules were mixed with 100 mL of medium A, sieved through a 400-µm mesh size sieve and immediately transferred into the anaerobic glove box (97% N_2_ and 3% H_2_ atmosphere). Thirdly, the mixture from step 2 was used to prepare a master-mix inoculum (1000 mL) with 10% (*v*/*v*) inoculum size. Then, the master-mix inoculum was homogenized by mixing at 250 rpm for 10 min, and 50 mL were transferred to serum bottles (200 mL) to start the experiment. The bottles were closed with butyl rubber stoppers and clamped with aluminum caps. The headspace was subsequently flushed and pressurized as described earlier (See Section 2.1). The batch cultures were fed once with the H_2_/CO_2_ gas mixture to verify the hydrogenotrophic activity of the inoculum before the inhibition experiments with BES were started. One bottle without inoculum served as a sterile control. Three bottles to monitor the residual biogas production from the inoculum alone were also included. Liquid and gas samples were taken after 3 h, 6 h, 24 h, 48 h, and 72 h. To test the effect of the medium composition, equal numbers of bottles were set up in the same manner as described before but with mineral medium B.

### 2.3. Inhibition Experiments with a Hydrogenotrophic Enrichment Culture

A hydrogenotrophic enrichment culture maintained in medium A was used as inoculum. The enrichment culture was fed daily with H_2_/CO_2_ (4:1) as described in our previous study [16] and transferred to fresh medium every 28 days. A 28-day-old culture was used as inoculum for the different experiments. The inoculum size was 10% (*v*/*v*). Experiments with and without BES were conducted in medium A and A1. To inhibit acetogenic bacteria, bottles with medium A were set up and supplemented with a cocktail of five different antibiotics (gentamicin (35 μg/mL), streptomycin (18 μg/mL), kanamycin (1 μg/mL), erythromycin (2 μg/mL), and vancomycin (60 μM)) as reported in a previous study [46]. This antibiotics cocktail has been used to isolate *Methanothrix* species [46,47] and does not inhibit methanogenic archaea [48]. The effect of yeast extract (0.20 g L^−1^) on the liquid products was tested in medium A2. Liquid and gas samples were taken after 3 h, 6 h, 24 h, 48 h, and 120 h.

### 2.4. Inhibition Experiments with Methanogenic Pure Cultures

Pure cultures of *Methanococcus maripaludis* DSM 14266, *Methanobacterium formicicum* DSM 1535, and *Methanosarcina barkeri* DSM 800 were purchased from the German Collection of Microorganisms and Cell Cultures (DSMZ) and used to investigate whether formic acid is produced during BES inhibition. Each methanogenic strain was initially cultured in the medium recommended by the DSMZ with H_2_/CO_2_ (4:1) as substrate. Subsequently, all strains were cultured in mineral medium C for at least three successive transfers with 10% (*v*/*v*) inoculum size to allow further comparison between the strains. Each strain was further maintained in medium C. Prior to the start of the BES inhibition experiment, a fresh pre-culture was prepared for each strain with 10% (*v*/*v*) inoculum from the latest maintenance culture. Both inhibited and non-inhibited cultures were started in five biological replicates with 10% (*v*/*v*) inoculum from the previous pre-culture and fed two times to ensure sufficient biomass growth before injecting BES to the bottles. Sterile control bottles contained the medium alone or medium plus BES. Liquid and gas samples were taken after 3 h, 6 h, 24 h, 48 h, and 72 h.

### 2.5. Analytical Methods

The pressure was measured with a high resolution manometer (LEO 5, Keller, Switzerland) in the same way as reported earlier [16]. One mL gas sample was withdrawn and the composition was analyzed via gas chromatography in a Perkin Elmer GC. Liquid samples were taken to analyze the volatile fatty acids in the liquid phase of every culture via high performance liquid chromatography (HPLC). In brief, 0.5 mL sample was withdrawn and centrifuged at 20,817× *g* and 4 °C for 10 min, subsequently filtered through a 0.2-μm membrane filter (13 mm; LABSOLUTE, Th. Geyer GmbH, Hamburg, Germany) and stored at −20 °C if not measured immediately. Detailed information about the GC and HPLC setup can be found in our previous article [16].

## 3. Results

### 3.1. Physiological Response of Anaerobic Granules to BES

**Figure 1:**
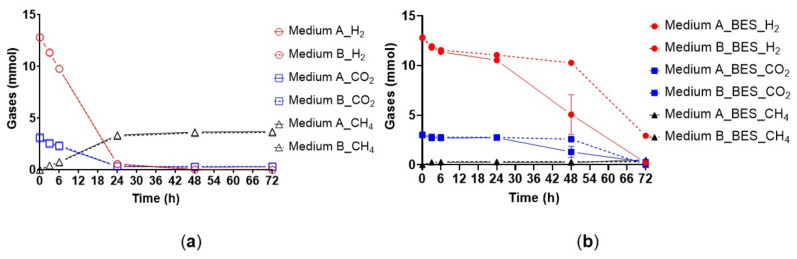
The gas phase for the inhibited and non-inhibited anaerobic granules in batch cultures with medium A and B. The bottles were pressurized solely with H_2_ (80%) and CO_2_ at ~2.2 bar during one batch cycle. All experiments were conducted in 200-mL serum bottles with 50 mL working volume. The error bars depict the standard deviation of the mean of *n* = 3 for non-inhibited cultures and *n* = 5 for inhibited cultures. When not visible the error bars are smaller than the symbol. Open symbols of panel (**a**) show non-inhibited cultures and filled symbols of panel (**b**) show BES-amended cultures with the following description: solid line (medium A), dashed line (medium B), circle (H_2_), square (CO_2_), and triangle (CH_4_).

Methane was produced without a lag-phase in all inoculated bioreactors for two consecutive batch feeding cycles prior to the addition of BES. The consumption of H_2_ and CO_2_ as well as the production of CH_4_ proceeded in a similar manner in the control bottles with media A and B (Figure 1a). The situation was different in the presence of BES: consumption of H_2_ and CO_2_ was delayed and CH_4_ production almost completely inhibited (Figure 1b). The CH_4_ amount after 72 h of incubation in the presence of BES was relatively low for medium A and medium B, respectively (Table 1). The gas consumption and production rates under different conditions are presented in Table 2. The detection of CH_4_ after 72 h in the inhibited bottles indicates that the inhibitory effect of BES on CH_4_ production was temporary for this complex microbial community under the conditions of our experiment.

**Figure 2:**
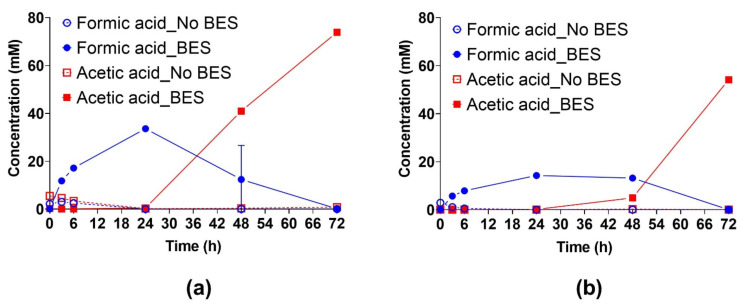
The effect of methanogenesis inhibition by BES (50 mM) on the production and consumption of formate and acetate by anaerobic granules in batch cultures with medium A (**a**) and B (**b**). Experimental conditions were as specified in Figure 1. The error bars depict the standard deviation of the mean of *n* = 3 for non-inhibited cultures and *n* = 5 for inhibited cultures. When not visible the error bars are smaller than the symbol. Blue circles: formate, red squares: acetate, filled symbols: BES added, open symbols: BES-free.

The formate concentrations in the non-inhibited cultures after 6 h were 2.49 ± 0.05 and 0.52 ± 0.02 mM for medium A and B, respectively. The small amounts of formate were rapidly consumed after 6 h in the cultures with both media (Figure 2). The complex microbial community of the anaerobic granules consumed H_2_/CO_2_ to produce CH_4_, and it produced and consumed formate but the formation of acetate was nearly negligible. Acetate was present in the beginning of the experiment up to 5.47 ± 0.10 and 0.04 ± 0.01 mM for medium A and B, respectively. The acetate concentration decreased to 0.18 ± 0.06 and 0.016 ± 0.01 mM after 24 h, and a slight increase after 72 h was observed.

The maximum formate concentrations in the BES-inhibited cultures after 24 h were 33.60 ± 0.41 mM and 14.25 ± 0.13 mM in medium A and B, respectively. The formate production rates were 1.23 mM h^−1^ (R^2^ = 0.903) and 0.51 mM h^−1^ (R^2^ = 0.862) in medium A and B, respectively. After 24 h formate was consumed rapidly at rates of 0.70 mM h^−1^ (R^2^ = 0.976) and 0.30 mM h^−1^ (R^2^ = 0.804) in medium A and B, respectively.

In the non-inhibited cultures, the acetate concentrations at the beginning of the experiment were 5.47 ± 0.10 and 0.03 ± 0.01 mM for medium A and B, respectively. After 72 h of incubation the acetate concentrations in medium A and B decreased to 0.80 ± 0.21 and 0.08 ± 0.004 mM, respectively (Figure 2). The BES-containing cultures produced acetate after 24 h of incubation, with a linear increase in medium A and with a longer lag-phase in medium B. In the presence of BES, acetate was produced at rates of 1.53 mM h^−1^ (R^2^ = 0.996) and 1.12 mM h^−1^ (R^2^ = 0.816) in medium A and B, respectively. The maximum acetate concentrations after 72 h were 73.86 ± 1.46 mM and 54.13 ± 0.68 mM in medium A and B, respectively. Acetate production was concomitant with formate consumption.

The concentrations of longer-chain carboxylates (propionate, *n*-butyrate, *iso*-butyrate, *n-*valerate, and *iso*-valerate) were also determined as shown in Figure S1. The maximum propionate concentrations in the BES-inhibited cultures were 0.62 ± 0.02 mM after 48 h and 0.34 ± 0.06 mM after 72 h in medium A and B, respectively. The *n-*butyrate concentrations after 72 h were 0.20 ± 0.02 mM and 0.25 ± 0.02 mM in medium A and B, respectively. The concentrations of other carboxylic acids in the BES-inhibited cultures were below 0.1 mM for both media. In the non-inhibited cultures, propionate reached 0.32 ± 0.01 mM after 6 h and decreased to 0.07 ± 0.03 mM after 72 h in medium A, whereas in medium B it reached up to 0.10 ± 0.12 mM after 24 h and disappeared after 72 h. The concentrations of other carboxylic acids in the non-inhibited cultures were below 0.2 mM for both media.

### 3.2. Physiological Response of the Hydrogenotrophic Enrichment Culture to BES

The hydrogenotrophic enrichment culture inhibited with BES produced formate and acetate in experiments conducted in media A and A1. Medium A2 (without yeast extract and vitamins) did not support microbial growth. The gas consumption and production rates are presented in Table 2. The antibiotics tested to inhibit homoacetogenic bacteria failed as was seen from acetate appearing in the broth (data not shown). The gas consumption and production rates of H_2_, CO_2_, and CH_4_ of the non-inhibited cultures in medium A were around two times higher than in medium A1 (Table 2). The cultures in medium A exposed to BES showed gas consumption rates of H_2_ and CO_2_ that were 22 and 10 times lower than those of the BES-free cultures (Table 2). Likewise, the inhibited cultures in medium A1 showed gas consumption rates (H_2_ and CO_2_) ≥ 40 times lower than non-inhibited cultures. The H_2_ and CO_2_ consumption rates of the BES-inhibited cultures in medium A were 5 and 10 times higher than the respective rates of the cultures in medium A1 (Table 2).

**Figure 3:**
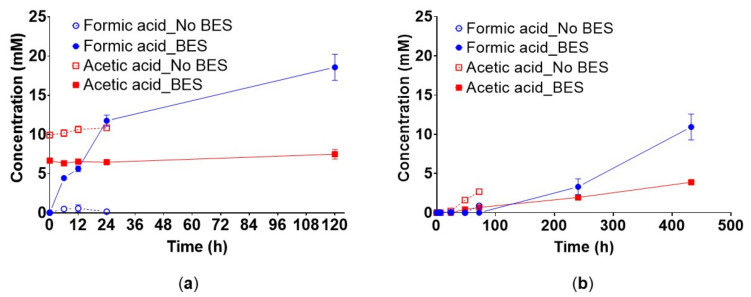
The effect of methanogenesis inhibition by BES (50 mM) on the production and consumption of formate and acetate by the hydrogenotrophic enrichment culture in medium A (**a**) and medium A1 (**b**). Experimental conditions were as specified in Figure 1. The error bars show the standard deviation of the mean of *n* = 3 and *n* = 4 for cultures in medium A and A1, respectively. When not visible the error bars are smaller than the symbol. Blue circles: formate, red squares: acetate, filled symbols: BES added, open symbols: BES-free.

The non-inhibited hydrogenotrophic enrichment cultures in medium A showed a peak of formate concentration of 0.588 ± 0.44 mM after 12 h. The final formate concentration of this culture decreased to 0.167 ± 0.03 mM after 24 h (Figure 3a). In case of the non-inhibited controls, data is provided only until 24 h because the H_2_/CO_2_ substrate was depleted.

In medium A in presence of BES and H_2_/CO_2_, formate concentration increased linearly up to 11.76 ± 0.71 mM after 24 h of incubation and further to 18.57 ± 1.68 mM after 120 h (Figure 3a). In medium A1 and in the presence of BES, formate started to be produced after 240 h of incubation (3.31 ± 1 mM) but acetate was detected after 48 h (0.42 ± 0.025 mM) (Figure 3b). Formate was also produced without BES, and after 72 h the maximum concentration was 0.89 ± 0.14 mM. Similarly, acetate accumulated in the non-inhibited cultures up to 2.7 ± 0.2 mM after 72 h (Figure 3b). It is noteworthy that when BES was added, the formate production rate was 0.47 mM h^−1^ (R^2^ = 0.975) and 0.03 mM h^−1^ (R^2^ = 0.966) in medium A and A1, respectively. The cultures in medium A produced remarkably more formate and in less time than in medium A1.

Acetate was already produced during the first two batch feeding cycles up to 9.93 ± 0.36 mM and 6.68 ± 0.29 mM in the non-inhibited and inhibited cultures, respectively, prior to starting the inhibition with BES in medium A. This indicates that yeast extract supported biomass growth and homoacetogenesis. The non-inhibited cultures in medium A produced acetate at 0.026 mM h^−1^ (R^2^ = 0.897) while formate was produced and consumed.

In the inhibited cultures in medium A, acetate was produced at 0.009 mM h^−1^ (R^2^ = 0.8708), which is much slower than the non-inhibited cultures, but formate was not consumed. In medium A1, acetate was detected after 24 h and 48 h in the non-inhibited and inhibited cultures, respectively. In the non-inhibited cultures, the maximum acetate concentration was 2.69 ± 0.198 mM after 72 h and until this time no consumption was observed. In the inhibited cultures, the maximum acetate concentration was 3.89 ± 0.236 mM after 18 d and consumption was not observed. The rates at which acetate was produced were 0.038 mM h^−1^ (R^2^ = 0.9524) and 0.008 mM h^−1^ (R^2^ = 0.9953) for the non-inhibited and inhibited cultures, respectively. Formate was not consumed concomitantly with acetate being produced.

The concentrations of longer-chain carboxylates (especially propionate and *n*-butyrate) are shown in Figure S2. The maximum propionate concentration in the BES-inhibited cultures was 0.26 ± 0.01 mM after 120 h and 0.28 ± 0.13 mM after 240 h in medium A and A1, respectively. The *n*-butyrate concentrations after 120 h were 0.20 ± 0.01 mM and 0.18 ± 0.02 mM in medium A and A1, respectively. In the non-inhibited cultures, propionate reached up to 0.25 ± 0.01 mM after 24 h in medium A, whereas in medium A1 it reached up to 0.22 ± 0.005 mM after 24 h and decreased to 0.19 ± 0.04 mM after 72 h. *Iso*-butyrate, *n*-valerate, and *iso*-valerate concentrations were below the detection limit in both BES-inhibited and non-inhibited cultures.

### 3.3. Physiological Response of Methanogenic Strains to BES

We tested the response of two pure culture methanogenic strains to BES when H_2_/CO_2_ was fed. Both methanogenic strains were successfully adapted to medium C. The H_2_ and CO_2_ consumption rates were comparable for both strains under non-inhibited conditions, however, the CH_4_ production rate of *M. maripaludis* was 2.5 times higher than that of *M. formicicum*.

The H_2_ conversion rates of *M. maripaludis* and *M. formicicum* cultures inhibited with BES were nine and four times lower than those of the respective non-inhibited cultures (Table 2). Likewise, the CO_2_ conversion rates were ten and two times lower for *M*. *maripaludis and M. formicicum* than those of the respective non-inhibited cultures (Table 2). For both strains, CH_4_ was not detected during the incubation period (72 h) in the presence of BES.

**Figure 4:**
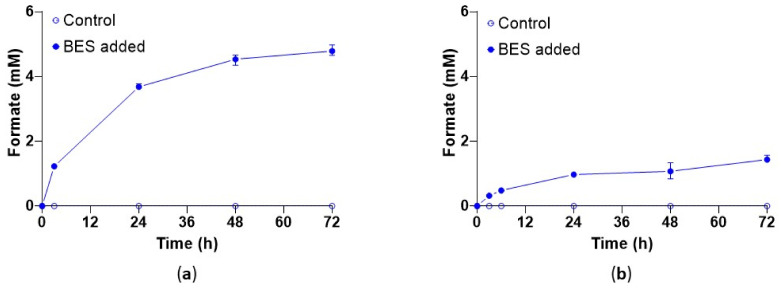
Response of the hydrogenotrophic methanogenic strains *Methanococcus maripaludis* (**a**) and *Methanobacterium formicicum* (**b**) to BES (50 mM) in medium C. The cultures were pressurized with H_2_ (80%) and CO_2_ at ~2.2 bar. The error bars depict the standard deviation of the mean of *n* = 5 (**a**) and *n* = 4 (**b**).

The two strictly hydrogenotrophic strains *M. maripaludis* and *M. formicicum* produced formate under methanogenesis inhibition with BES. The production of formate was almost linear during the first 24 h for both strains (Figure 4). At the end of the incubation, 4.2 mM and 1.8 mM were produced by *M. maripaludis* and *M. formicicum*, respectively. Formate was not detected under non-inhibited conditions.

## 4. Discussion

Hydrogenotrophic methanogenesis is the key pathway in biomethanation of H_2_/CO_2_. However, other metabolic routes such as homoacetogenesis followed by acetoclastic methanogenesis can take place in complex and enrichment cultures. BES is a common methanogenesis inhibitor [28] and useful to study alternative hydrogenotrophic reactions such as homoacetogenesis [27,49]. BES has been used in pure cultures of methanogens as well as in complex communities [32,33,34,35,50]. While a previous study focused on formate production in pure and mixed cultures [33] and a more recent study aimed at using BES to produce acetate [17], neither study looked at both formate and acetate production during inhibition of methanogenesis. In our study, subjecting crushed anaerobic granules, a hydrogenotrophic enrichment culture, and methanogenic pure cultures to BES resulted in different levels of formate production and, in case of the mixed cultures, acetate production. Inhibition of bacterial activity by antibiotics failed, which could be due to the concentration of the antibiotics mixture or the presence of resistant bacteria in the community, thus we could not narrow down how much the archaeal community alone contributed to formate production. All BES-free cultures were effective at consuming the supplied H_2_/CO_2_ independently of the medium used. The methane production rates of the non-inhibited anaerobic granules in medium A and B were two and three times lower than those observed in our previous study using a similar inoculum in medium A [45], but they were comparable to the rates reported from other studies in a recent review [51]. The previously reported methane production rate of the hydrogenotrophic enrichment culture [16] was confirmed. The pure cultures tested in this study showed similar methane production rates as reported in a recent study screening a massive number of methanogenic strains [10].

The situation was different when BES was added to the cultures, as the H_2_ and CO_2_ consumption rates dramatically decreased in all cultures. Whereas the hydrogenotrophic enrichment culture and the pure cultures were inhibited completely, the anaerobic granules produced some methane at the end of the cultivation period, which indicates that the inhibitory effect of BES on the methane production was temporary for this complex microbial community and under the conditions of our experiment. This is in agreement with a former study with anaerobic granules and BES as methanogenesis inhibitor [33]. It is possible that complex communities degrade BES as observed in bioelectrochemical systems [52]. Another study established enrichment cultures with BES as inhibitor and isolated a *Desulfovibrio* sp. strain that was capable of BES degradation [53]. To this end, the traces of CH_4_ that were produced by the anaerobic granules in our study might be explained by adaptation of the microbial community to BES or its degradation. We have previously investigated the community composition of anaerobic granules sampled from the same wastewater treatment plant and the hydrogenotrophic enrichment culture. The dominant methanogens in the anaerobic granules were *Methanobacterium* and *Methanothrix* [45], whereas the hydrogenotrophic enrichment culture was dominated by *Methanobacterium* and *Methanoculleus* [16]. The dominant bacterial orders in the anaerobic granules were *Anaerolineales*, *Bacteroidales, Eubacteriales* and several other clostridial orders, *Nitrospirales, Syntrophobacterales, Desulfuromonadales, Micrococcales, Synergistales*, *Candidatus* Fermentibacterales, *Spirochaetales, Marinilabiales, Thermotogales, Campylobacterales*, and unclassified members of the *Bacteroidetes, Chloroflexi, Cloacimonetes*, and *Verrucomicrobia* [45]. The hydrogenotrophic enrichment culture was less diverse and comprised the bacterial orders *Eubacteriales, Bacteroidales, Thermoanaerobacterales,* and unclassified *Firmicutes* [16]. A major difference between the mixed cultures used in this study was the presence of an acetoclastic methanogen in the anaerobic granules. Assuming that homoacetogenic bacteria were present in the inoculum and considering that *Methanothrix* was abundant in the anaerobic granules as previously reported [45], H_2_/CO_2_ could be channeled to acetate and subsequently converted to methane by acetoclastic methanogens. The situation was different for the hydrogenotrophic enrichment culture because acetoclastic methanogens were absent [16]. Here, methane production solely relies on hydrogenotrophic methanogenesis and acetate could be only degraded by syntrophic acetate oxidation, which requires the concerted action of syntrophic acetate-degrading bacteria (performing the Wood-Ljungdahl pathway in the opposite direction than during homoacetogenesis) and hydrogenotrophic methanogens. Hence, inhibiting hydrogenotrophic methanogenesis and keeping a high hydrogen partial pressure would make syntrophic acetate oxidation thermodynamically unfavorable and boost homoacetogenesis.

In a recent study with the hydrogenotrophic enrichment culture, we showed that formate was produced and consumed during biomethanation of H_2_/CO_2_ without any methanogenesis inhibitor [16]. In the present study, formate was notably detected in inhibited cultures of crushed anaerobic granules and the hydrogenotrophic enrichment culture but to a lesser extent also in inhibitor-free cultures, which is in line with a previous study [33] despite differences in the experimental setups (only 2.5 mM BES, granules were not crushed, differences in the reactor setup). Other studies have subjected mixed cultures to BES to study the physiology [34] or the production of organic acids [32] but formate was not detected in the liquid phase when H_2_, CO_2_ or CO were supplied as substrate, likely due to the sampling intervals. To clarify if formate is an intermediate of hydrogenotrophic growth, we assembled cultures using the same medium as Omar and colleagues [17] but sampled at shorter time intervals and found that formate was indeed an intermediate produced in BES-inhibited mixed cultures prior to acetate production. Formate was not detected in the BES-free pure cultures, which could be explained by a turnover that was faster than our sampling intervals, or formate was not excreted or its concentration was below our detection limit. The pure methanogenic cultures subjected to BES accumulated formate in the medium as reported in previous studies [33,54,55]. All cultures inhibited with BES produced formate rapidly at the beginning of the incubations except for the hydrogenotrophic enrichment culture in medium A1 (mineral medium, pH 9), which was the only one showing a lag-phase.

Besides formate and acetate, small amounts of longer-chain carboxylates with three to five carbon atoms were produced by the mixed cultures. These products might be attributed to the activity of homoacetogenic bacteria, which can produce butyrate in small proportions besides the main products acetate and ethanol [56], and the concerted action of homoacetogenic and propionigenic bacteria [57]. Additionally, acetate can be elongated to butyrate or propionate to valerate by reverse β-oxidation, an alternative electron sink in anaerobic consortia especially when methanogenesis is inhibited and when the syntrophic oxidation of carboxylates by proton-reducing bacteria becomes thermodynamically unfavorable due to high hydrogen partial pressure [58]. Thus, chain elongation processes in anaerobic mixed cultures are supported by feeding hydrogen [32]. The variety and concentrations of C3-C5 products in the anaerobic granules culture were higher than those in the hydrogenotrophic enrichment culture. These differences could be explained by the higher bacterial diversity of the anaerobic granules compared with the hydrogenotrophic enrichment culture, and consequently the presence of more different metabolic pathways. Our results are in line with the study of Omar et al. who found the same carboxylates being produced when methanogenic sludge was subjected to BES treatment [17].

When performing biomethanation of H_2_/CO_2_ with pure strains or mixed cultures, formate is an intermediate in the process. The formate production observed in the BES-free mixed cultures in our experiments could be attributed to the H_2_/CO_2_ metabolism of either methanogens or homoacetogens considering that both types of microorganisms can transiently synthesize formate, yet differences in the molecular mechanisms of each pathway exist [22]. The acetyl-CoA pathway is used by acetogens and methanogens to derive the carbon and energy needed when growing on H_2_/CO_2_. In acetogens and methanogens, CO_2_ is reduced to formate and CO as the first steps in the acetyl-CoA pathway [22]. The reduction of CO_2_ for the methyl branch is different in methanogens and acetogens but conserved for the carbonyl branch [22,59,60]. The energy investment in formate-generating enzymes of acetogens and methanogens is strikingly different: while acetogens invest one ATP for formate fixation, methanogens bypass this energy cost [22]. Interestingly, acetogens generate one ATP in the last step of acetate formation from acetyl-CoA but no ATP is produced by methanogens in methane formation [22].

Previous studies that used BES to inhibit methanogens in mixed cultures observed acetate production from H_2_/CO_2_ [17,34]. The maximum acetate concentrations in our study were four and three times higher than those observed in a previous study [17]. Although we used the same medium as Omar and colleagues [17] (medium B in our study), our results indicated higher acetate production, which could be related to the inoculum source, reactor setup, or inoculum preparation (we used crushed granules sieved through a finer mesh size of 400 µm). Interestingly, the time required to produce significantly higher concentration of acetate was shorter in our study and one could argue that this was due to the higher microbial biomass (biocatalyst) available in our setup. BES inhibits methanogenesis and thus steers the carbon and electron flow towards acetate production via homoacetogenesis. Here, we argue that formate is an important accumulating intermediate under BES-inhibited conditions based on the following reasons: (a) simultaneous consumption of formate and production of acetate; (b) formate is first produced by either homoacetogenic bacteria or methanogens with the metabolic potential to use H_2_/CO_2_ or formate; (c) under methanogenesis inhibition, formate is subsequently utilized by the bacterial community to produce acetate. However, it cannot be ruled out that formate is interconverted to H_2_/CO_2_.

Methanogens can also produce formate from H_2_/CO_2_ with chloroform or ethanol as inhibitors [33]. Previous studies using BES as a methanogenesis inhibitor measured volatile fatty acids produced after 48 h [17,61]. On the basis of our results we suggest that the sampling interval should be short enough to capture the profile of intermediates and final products in the liquid phase according to the aim of the study. BES is an analogue of methyl-coenzyme M (Co-M) and competes with this coenzyme in the methanogenic pathway, thus, resulting in the inhibition of methane production [52,62]. Methanogenesis inhibition with BES alters the activity and community structure of methanogens and was shown to increase the copy number of formyltetrahydrofolate synthetase (*fhs*) genes, thus stimulating homoacetogenesis [63]. Different scenarios are conceivable during methanogenesis inhibition with BES. In the scenario without inhibition with mixed cultures, methane and acetate can be produced concomitantly from H_2_/CO_2_ depending on the H_2_ partial pressure wherein formate is an intermediate during their production as described by Lemaire et al. [22]. In the scenario of complete inhibition to study homoacetogenesis in environmental or sludge samples, formate is an intermediate that is produced by methanogens or acetogens prior to acetate production. In a scenario of partial inhibition of methanogenesis, formate in the bulk is produced by methanogens and acetogens and can be channeled to methane and acetate.

## 5. Conclusions

This study showed with pure methanogenic strains, a highly enriched hydrogenotrophic community, and anaerobic granules from a wastewater treatment system that formate is an important intermediate of H_2_/CO_2_ metabolism during methanogenesis inhibition with BES. It is suggested that when studying homoacetogenesis under methanogenesis inhibition with BES, formate accumulation occurs before acetate production, which has been frequently neglected. While BES (50 mM) exerted a strong inhibition of pure methanogenic strains and the enriched community, the inhibition of the complex community of anaerobic granules was transient. Furthermore, in the absence of the methanogenesis inhibitor, formate was also produced from H_2_/CO_2_, which could be explained as a physiological feature of methanogens or homoacetogens with the metabolic potential of using H_2_/CO_2_ and formate. This shows that formate synthesis is a concomitant reaction taking place in processes both in case of ex situ or in situ biomethanation of H_2_/CO_2_.

## Figures and Tables

**Table 1:** Major features of the mineral media used for the tested cultures.

Type of Medium	Remarks	Anaerobic Granules	Hydrogenotrophic Enrichment Culture	Methanogenic Pure Cultures
**Medium A**	− With yeast extract (0.2 g L^−1^) − Without vitamins − Replicates with BES − Replicates without BES			
	
		5	3	
		3	3	
**Medium A1**	− Without yeast extract − With vitamins − Replicates with BES − Replicates without BES			

			4	
			4	
**Medium A2**	− Without yeast extract − Without vitamins − Replicates without BES only			

			3	
**Medium B**	− Without yeast extract − With vitamins − Replicates with BES − Replicates without BES			

		5		
		3		
**Medium C**	− Without yeast extract − With vitamins − Replicates with BES − Replicates without BES			

				4 ^a^ and 5 ^b^
				4 ^a^ and 5 ^b^

Note: The superscript letters indicate the type of methanogenic strain ^a^ = *M. maripaludis* and ^b^ = *M. formicicum*.

**Table 2:** Gas consumption (H_2_, CO_2_) and CH_4_ production rates under different experimental conditions.

Culture Type	BES (50 mM)	H_2_ (mmol h^−1^)	CO_2_ (mmol h^−1^)	CH_4_ (mmol h^−1^)
Crushed anaerobic granules in medium A	Free ^3^	0.51 ± 0.0006	0.11 ± 0.0009	0.14 ± 0.0010
	Added ^5^	0.08 ± 0.0023	0.05 ± 0.0057	0.04 ± 0.0004
Crushed anaerobic granules in medium B	Free ^3^	0.51 ± 0.0014	0.11 ± 0.0003	0.14 ± 0.0008
	Added ^5^	0.06 ± 0.0020	0.04 ± 0.0104	0.05 ± 0.0009
Hydrogenotrophic enrichment culture in medium A	Free ^3^	0.44 ± 0.0040	0.10 ± 0.0017	0.11± 0.0011
	Added ^3^	0.02 ± 0.0022	0.01± 0.0023	-
Hydrogenotrophic enrichment culture in medium A1	Free ^4^	0.19 ± 0.0035	0.04 ± 0.0008	0.05 ± 0.0016
	Added ^4^	0.004 ± 0.0005	0.001 ± 0.0002	-
*M. maripaludis* in medium C	Free ^4^	0.09 ± 0.001	0.02 ± 0.0004	0.05 ± 0.001
	Added ^4^	0.01 ± 0.001	0.002 ± 0.0004	-
*M. formicicum* in medium C	Free ^5^	0.08 ± 0.0012	0.02 ± 0.0007	0.02 ± 0.0003
	Added ^5^	0.02 ± 0.0004	0.01 ± 0.0004	-

Note: The superscript number indicates the number of biological replicates. The mean and standard deviation are presented.

## Data Availability

The data presented in this study are available in this article and its Supplementary Materials.

## Supplementary Materials

The following supporting information can be downloaded at: https://www.mdpi.com/article/10.3390/microorganisms10020355/s1, Table S1: Composition of the medium component 1 for media A, A1 and A2; Table S2: Stock solutions used to supplement media A, A1 and A2; Table S3: Composition of the stock solutions for media A, A1 and A2 [64]; Table S4: Composition of medium B [65]; Table S5: Composition of the medium component 1 for medium C [66]; Table S6: Stock solutions used to supplement medium C; Table S7: Composition of the vitamin solution for medium C; Figure S1: Concentrations of longer-chain carboxylates in the anaerobic granules culture in medium A and B; Figure S2: Concentrations of longer-chain carboxylates in the hydrogenotrophic enrichment culture in medium A and A1.
