# Supplementary material for: Physiological Effects of 2-Bromoethanesulfonate on Hydrogenotrophic Pure and Mixed Cultures
Source: Microorganisms. 2022 Feb 3;10(2):355. doi: 10.3390/microorganisms10020355 (PMC8877471; doi:10.3390/microorganisms10020355)
Supplement: Supplementary file 1 [file microorganisms-10-00355-s001.zip › microorganisms-1537532-supplementary.pdf]

microorganisms

Physiological effects of 2-bromoethanesulfonate on hydrogenotrophic pure and mixed cultures

Washington Logroño, Marcell Nikolausz, Hauke Harms and Sabine Kleinsteuber*

Department of Environmental Microbiology, Helmholtz Centre for Environmental Research – UFZ, 04318 Leipzig, Germany; washington.logrono@ufz.de (W.L.); marcell.nikolausz@ufz.de (M.N.); hauke.harms@ufz.de (H.H.)
*Correspondence: sabine.kleinsteuber@ufz.de (S.K.)

Supplementary materials

Table S1. Composition of the medium component 1 for media A, A1 and A2
Table S2. Stock solutions used to supplement media A, A1 and A2
Table S3. Composition of the stock solutions for media A, A1 and A2
Table S4. Composition of medium B
Table S5. Composition of the medium component 1 for medium C
Table S6. Stock solutions used to supplement medium C
Table S7. Composition of the vitamin solution for medium C

Table S1. Composition of the medium component 1 for media A, A1 and A2. The anoxic and sterile component 1 was supplemented with stock solutions (**Table S2**) according to the requirements of media A, A1 or A2. Composition of the stock solutions is provided in **Table S3**.

**Compound**	**Final concentration (g L^{-1})**		
	Medium A	Medium A1	Medium A2
NH_4Cl	0.5	0.5	0.5
KH_2PO_4	0.2	0.2	0.2
$MgCl_2 \times 6\ H_2O$	0.1	0.1	0.1
KCl	0.2	0.2	0.2
NaCl	2.0	2.0	2.0
Yeast extract	0.2	-	-
Resazurin	0.0005	0.0005	0.0005
Trace elements SL10 (mL L^{-1})	1	1	1

Note: Component 1 (850 mL) was made anoxic by stirring in the anaerobic chamber (97% N_2, 3% H_2) for 45 min. Sterilization was done by autoclaving at 121°C for 20 min.

Table S2. Stock solutions used to supplement media A, A1 and A2

**Stock solutions**	**Volume added (mL L^{-1})**		
	Medium A	Medium A1	Medium A2
Selenite-tungstate solution DSMZ 385 (1:4 diluted)	4	4	4
Na_2CO_3 (29.41 g L^{-1})	34	34	34
$NaHCO_3$ (76.00 g L^{-1})	100	100	100
Cysteine-HCl (30.00 g L^{-1})	12	12	12
Vitamin solution	-	1	-

Note: Every stock solution was made anoxic by stirring in the anaerobic chamber for 30 min.

Table S3. Composition of the stock solutions for media A, A1, and A2

Component	Concentration (mg L^{-1})
Trace elements SL10 (DSMZ medium 320)	
$FeCl_2 \times 4\ H_2O$	1500
$ZnCl_2$	70
$MnCl_2 \times 4\ H_2O$	100
H_3BO_3	6
$CoCl_2 \times 6\ H_2O$	190
$CuCl_2 \times 2\ H_2O$	2
$NiCl_2 \times 6\ H_2O$	24
$Na_2MoO_4 \times 2\ H_2O$	36
Selenite-tungstate solution (DSMZ medium 385)	
NaOH	500
Na_2SeO_3 x 5 H_2O	3.0
Na_2WO_4 x 2 H_2O	4.0
Vitamin solution [1]	
Biotin	20
Folic acid	20
Pyridoxine	100
Thiamine	50
Riboflavin	50
Nicotinic acid	50
Calcium pantothenate	50
Vitamin B12	20
p-Aminobenzoate	80
Lipoic acid	50

Note: For preparing SL10, $FeCl_2$ was first dissolved in HCl (25%, 10 mL L^{-1}) and then diluted in water. Subsequently, other salts were added and dissolved.

Table S4. Composition of medium B [2]

Stock A	Concentration (g L^{-1})
NH_4Cl	100
NaCl	10
$MgCl_2 \times 6\ H_2O$	10
$CaCl_2 \times 2\ H_2O$	5
**Stock B**	
KH_2PO_4 3 H_2O	200
**Stock C**	
Resazurin	0.5
**Stock D (Trace-metal and selenite solution)**	
$FeCl_2 \times 4\ H_2O$	2
H_3BO_3	0.05
$ZnCl_2$	0.05
$CuCl_2 \times 2\ H_2O$	0.038
$MnCl_2 \times 4\ H_2O$	0.05
$(NH_4)_6Mo_7O_{24} \times 4\ H_2O$	0.05
$AlCl_3$	0.05
$CoCl_2$ 6 × H_2O	0.05

$NiCl_2 \cdot 6 \times H_2O$	0.092
Ethylenediaminetetraacetate	0.5
Concentrated HCl (mL)	1
$Na_2SeO_3 \times 5\ H_2O$	0.1
**Stock E (Vitamins)**	**Concentration (mg L^{-1})**
Biotin	2
Folic acid	2
Pyridoxine acid	10
Riboflavin	5
Thiamine hydrochloride	5
Vitamin B12	0.1
Nicotinic acid	5
Calcium pantothenate	5
***p*-Aminobenzoate**	5
Lipoic acid	5

Note: The stock solutions were added to 975 mL distilled water in the following volumes: (**A**), 10 mL; (**B**), 2 mL; (**C**), 1 mL; (**D**), 1 mL and (**E**), 1 mL. The mixture was gassed with N_2/CO_2 (80-20%) mixture to maintain a neutral pH. Cysteine hydrochloride, 0.5 g and $NaHCO_3$, 2.6 g dissolved in 10 mL distilled water were added. The medium was then dispensed to serum bottles. The medium was supplemented with $Na_2S \times 9H_2O$ to a final concentration of 0.025% before inoculation.

Table S5. Composition of the medium component 1 for medium C [3]. The anoxic and sterile component 1 was supplemented with stock solutions as indicated in **Table S6**.

Compound	Final concentration (g L^{-1})
KCl	0.34
$MgCl_2$ x 6 H_2O	4.00
NH_4Cl	0.25
$CaCl_2$ x 2 H_2O	0.14
Na-K PO_4 (mL L^{-1})	1
NaCl	18
Trace elements SL10 (DSMZ medium 320) (mL L^{-1})	1
Selenite-tungstate solution (DSMZ medium 385) (mL L^{-1})	1

Note: Component 1 (960 mL) was made anoxic by stirring in the anaerobic chamber (97% N_2, 3% H_2) for 45 min. Sterilization was done by autoclaving at 121°C for 20 min. The composition of SL10 and selenite-tungstate solution is provided in **Table S3**.

Table S6. Stock solutions used to supplement medium C. The composition of the vitamin solution is provided in **Table S7**.

Stock solutions	Volume added (mL L^{-1})
$MgSO_4 \times 7\ H_2O$ (3.45 g / 100 mL)<br>$Fe(NH_4)_2(SO_4)_2 \times 6\ H_2O$ (20 mg / 100 mL)	10
$NaHCO_3$ (30 mM)	30
Na_2HPO_4 (75 μM)<br>KH_2PO_4 (75 μM)	1
Vitamin solution (DSMZ media 141 and 384)	1

Table S7. Composition of the vitamin solution for medium C (DSMZ media 141 und 384)

Component	Concentration (mg L^{-1})
Biotin	2
Folic acid	2
Pyridoxine - HCl	10
Thiamine - HCl	5
Riboflavin	5
Nicotinic acid	5
Calcium pantothenate	5
Vitamin B12	0.1
p-Aminobenzoate	5
Lipoic acid	5

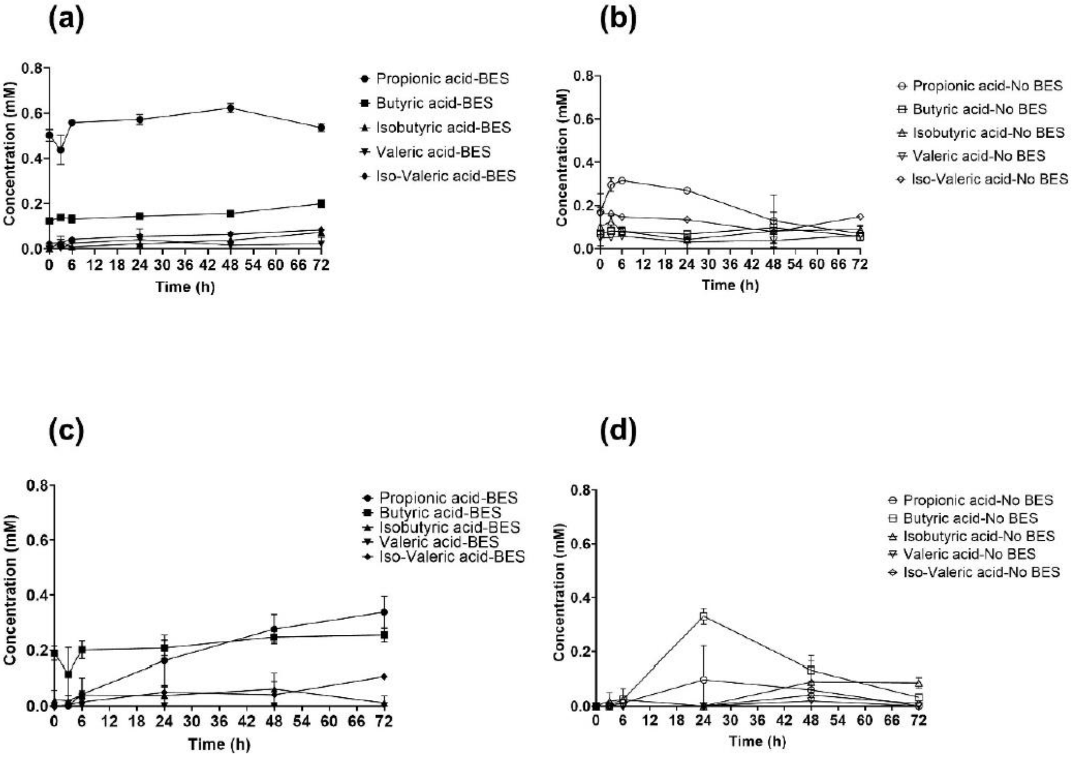

Figure S1. Concentrations of longer-chain carboxylates in the anaerobic granules culture in medium A and B. BES-inhibited culture in medium A (a), non-inhibited culture in medium A (b), BES-inhibited culture in medium B (c), non-inhibited culture in medium B (d). The bottles were pressurized with H_2 (80%) and CO_2 at ~2.2 bar during one batch cycle. All experiments were conducted in 200-mL serum bottles with 50 mL working volume. The error bars depict the standard deviation of the mean of n = 3 for non-inhibited cultures and n = 5 for inhibited cultures. When not visible the error bars are smaller than the symbol. Filled symbols: BES added, open symbols: BES-free.

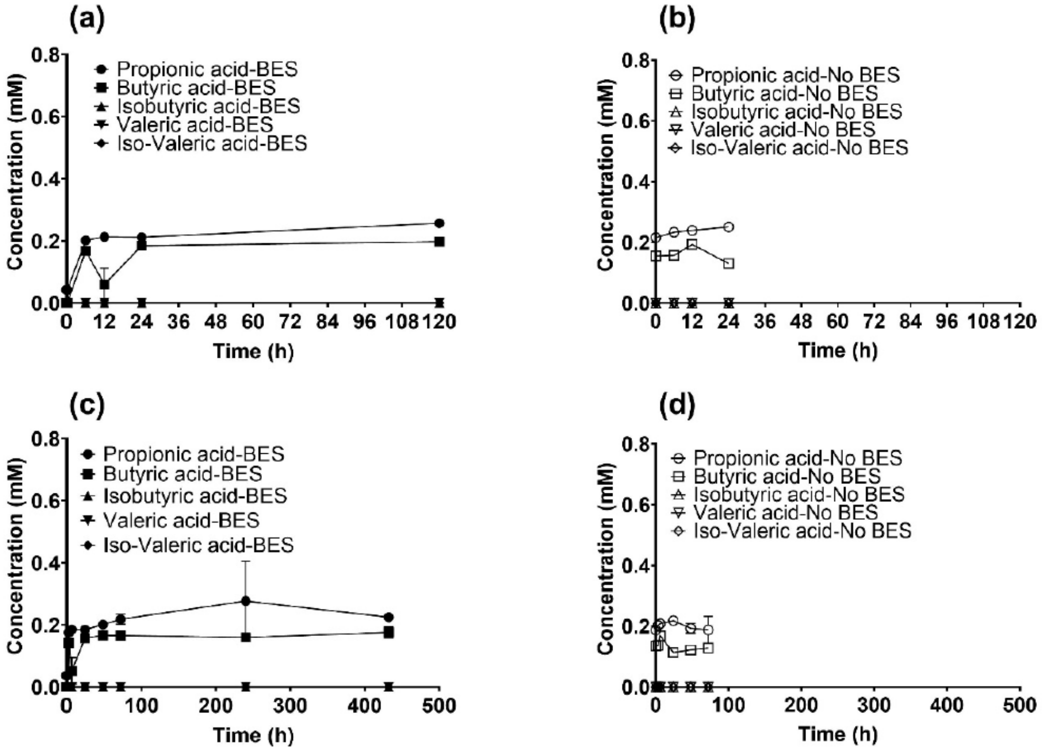

Figure S2. Concentrations of longer-chain carboxylates in the hydrogenotrophic enrichment culture in medium A and A1. BES-inhibited culture in medium A (a), non-inhibited culture in medium A (b), BES-inhibited culture in medium A1 (c), non-inhibited culture in medium A1 (d). Experimental conditions were as specified in Figure S1. The error bars show the standard deviation of the mean of n = 3 and n = 4 for cultures in medium A and A1, respectively. When not visible the error bars are smaller than the symbol. Filled symbols: BES added, open symbols: BES-free.

References

1. Bonk, F.; Popp, D.; Weinrich, S.; Sträuber, H.; Becker, D.; Kleinsteuber, S.; Harms, H.; Centler, F. Determination of Microbial Maintenance in Acetogenesis and Methanogenesis by Experimental and Modeling Techniques. *Frontiers in Microbiology* **2019**, *10*, 1–13, doi:10.3389/fmicb.2019.00166.

2. Omar, B.; Abou-shanab, R.; El-gammal, M.; Fotidis, I.A.; Kougias, P.G.; Zhang, Y.; Angelidaki, I. Simultaneous Biogas Upgrading and Biochemicals Production Using Anaerobic Bacterial Mixed Cultures. *Water Research* **2018**, *142*, 86–95, doi:10.1016/j.watres.2018.05.049.

3. Deutzmann, J.S.; Spormann, A.M. Enhanced Microbial Electrosynthesis by Using Defined Co-Cultures. *ISME Journal* **2017**, *11*, 704–714, doi:10.1038/ismej.2016.149.
